# Translating the 2024 ACR lupus nephritis guidelines to pediatric practice

**DOI:** 10.3389/fped.2026.1970903

**Published:** 2026-10-01

**Authors:** Minh Dien Duong, Quang Le, Siddharth Shah

**Affiliations:** 1 Division of Pediatric Nephrology, Norton Children’s Hospital, University of Louisville School of Medicine, Louisville, KY, United States; 2 Department of Medicine, University of Missouri, Columbia, MO, United States

**Keywords:** American college of rheumatology, belimumab, calcineurin inhibitor, childhood-onset systemic lupus erythematosus, clinical practice guideline, glucocorticoids, lupus nephritis, triple therapy

## Abstract

Lupus nephritis (LN) is more common and often more aggressive in children than in adults, yet most randomized trials informing current LN management have enrolled adults. The 2024 American College of Rheumatology (ACR) guideline is the first ACR guideline on lupus nephritis to explicitly state that its recommendations apply to both adults and children**,** although its evidence base remains largely adult-derived. This review uses the 2024 ACR guideline as an organizing framework, cross-referenced with the 2024 KDIGO and 2025 EULAR recommendations, to examine which aspects of LN management translate effectively to pediatric practice and which require caution. We review the diagnostic pathway, including screening, kidney biopsy, and ISN/RPS classification, as well as therapeutic strategies, including the shift from sequential dual therapy to upfront triple therapy, response targets, class-specific treatment, and selection between belimumab and calcineurin inhibitors. We then examine areas where pediatric evidence remains limited, particularly glucocorticoid dosing, long-term biologic safety, fertility and growth, and transition to adult care. While the fundamental principles of LN management are largely age-agnostic, many quantitative recommendations are not. Steroid dosing, long-term treatment exposure, and developmental consequences remain important pediatric-specific considerations that current guidelines cannot fully address. We conclude by highlighting the pediatric evidence available to date and the need for dedicated pediatric trials to replace adult-derived extrapolation with child-specific evidence.

## 1 Introduction

Treating children with lupus nephritis (LN) has historically relied largely on adult guidelines. Compared with the 2012 guideline, the 2024 ACR guideline states that recommendations for the screening, treatment, and management of lupus nephritis apply to both adults and children, with pediatric considerations incorporated throughout the main document rather than in a separate appendix (1). This represents an important step forward, but it also carries limitations. The evidence behind the 2024 ACR document, like the 2024 KDIGO guideline (2) and the 2025 EULAR update on systemic lupus erythematosus (SLE) with kidney involvement (3), comes almost entirely from adult randomized controlled trials. Consequently, when adult-derived recommendations including steroid dosing, monitoring intervals, and cumulative exposure to glucocorticoids and immunosuppressive therapies are applied to children, clinical decisions often rely more on expert judgment than on pediatric-specific evidence.

This review takes the practical view of pediatric nephrologists and rheumatologists. We conducted a focused search of PubMed/MEDLINE and of relevant guideline and professional society websites, covering literature published from January 2012 (the year of the preceding ACR guideline) through August 2026, for literature on lupus nephritis, with an emphasis on the 2024 ACR guideline and pediatric studies. Pediatric evidence was prioritized when available, particularly when it differed from the predominantly adult evidence. As this is a narrative review, the search was not systematic or exhaustive. We use the 2024 ACR recommendations as a framework to navigate the diagnostic and therapeutic approach to proliferative and membranous LN in children. At each step, we consider whether adult-derived recommendations can be appropriately applied to pediatric patients. Some concepts translate well, particularly the emphasis on early triple therapy and reducing cumulative glucocorticoid exposure, which is very important in children. However, other areas remain less certain, especially glucocorticoid dosing strategies and the long-term safety of therapies that children may require for many years. Pediatric LN data remain limited, and clinical decisions in these areas often rely on individual clinician judgment. Where pediatric-specific European recommendations exist, namely the SHARE initiative for childhood-onset SLE and childhood LN (4, 5), we use them as a comparator for how a pediatric-first document differs from an adult-first one.

We do not try to re-summarize the guidelines, which are freely available and well written. The aim is to read them through a pediatric lens and to make the translation gaps explicit, so they can be managed deliberately instead of being discovered at the bedside.

## 2 Pediatric lupus nephritis: what makes it different?

About 10%–20% of patients with SLE are diagnosed during childhood (6, 7). Compared with adult-onset SLE, childhood-onset SLE (cSLE) is generally more aggressive, has a stronger genetic contribution, and involves more organ systems (8). Kidney involvement is also more common in children, reported in approximately 30%–70% of those with childhood-onset SLE compared with 20%–40% of adults, with the wide range reflecting differences in cohort ascertainment, ethnicity, and biopsy practice, and it often develops early, at or near the time of SLE diagnosis (7, 9–12) (Table 1).

**Table 1:** Clinical features and reported renal outcomes of childhood- and adult-onset lupus nephritis (6–12).

Features	Childhood-onset LN	Adult-onset LN
Frequency of LN	30-70%	20-40%
Timing of LN	Often early; up to 93% reported within the first 2 years of SLE diagnosis	Variable
Complete remission rate with standard therapy	40-60%	60%
Renal flare rate	25-60%	30-40%
Progression to CKD and ESKD	Advanced CKD: 5-20% ESKD: 10-20%	CKD stage 3–5: 4.3-6.7% ESKD: ∼22% at 15 years

Values represent approximate ranges reported across individual pediatric and adult cohorts and are not pooled estimates. Studies differed in patient populations, treatment regimens, follow-up duration, and definitions of renal remission, renal flare, CKD, and ESKD. Therefore, these estimates are descriptive and should not be interpreted as directly comparable between childhood- and adult-onset LN.

Children with proliferative lupus nephritis (LN) achieve complete renal remission in only about 40%–60% of cases with standard therapy, while renal flares occur in approximately 25%–60% of patients (7, 9, 12). Recurrent LN flares contribute to cumulative kidney damage and progression to chronic kidney disease (CKD) (10, 11). Across cohorts, approximately 5%–20% of children develop advanced CKD, and 10%–20% eventually progress to end stage kidney disease (ESKD) (9, 10, 12). Despite advances in immunosuppressive therapy, the incidence of ESKD in pediatric LN has changed little, highlighting the need for earlier risk stratification and more effective, better-tolerated treatment strategies (10, 11).

Children with LN have substantially higher mortality than the general pediatric population, with infection and cardiovascular disease becoming leading causes of death, particularly after progression to ESKD (11). The time horizon is particularly important in pediatric LN. Children diagnosed with LN may live for many decades with both the consequences of the disease and cumulative treatment-related toxicity (1, 11).

## 3 Diagnosis of lupus nephritis

### 3.1 Screening for lupus nephritis

For patients with SLE without known kidney disease, the 2024 ACR guideline strongly recommends screening for proteinuria every 6–12 months, or sooner during an extra-renal lupus flare and annual testing is considered adequate for long-standing, mild, inactive disease (1). Although this recommendation applies to both adults and children, the optimal screening interval in children remains uncertain because pediatric-specific evidence is limited. Given the higher frequency and earlier onset of LN in childhood-onset SLE, a more vigilant approach may be appropriate. The European SHARE recommendations similarly emphasize regular renal surveillance in children with SLE (4, 5). More recently, the 2026 British Society for Rheumatology (BSR) guideline provides a more pediatric-specific framework: patients with active SLE should be reviewed every 1–3 months, with urinalysis and proteinuria assessment with urine protein-to-creatinine ratio (UPCR) included in routine monitoring, while children with stable low disease activity or remission should be monitored at least every 6 months (13). Thus, rather than interpreting the ACR 6–12-month interval as a fixed schedule for every child, pediatric practice should individualize surveillance according to disease activity, duration of remission, age, prior renal involvement, serologic activity, and other risk factors.

### 3.2 Indications for a kidney biopsy

The 2024 ACR guideline states as a Good Practice Statement that prompt kidney biopsy should be performed when LN is suspected, unless contraindicated or not feasible. Kidney biopsy is essential for establishing diagnosis and guiding treatment (1). The guideline conditionally recommends kidney biopsy for proteinuria with UPCR >0.5 mg/mg (first morning urine) and/or otherwise unexplained impaired kidney function and conditionally recommends repeat biopsy for a suspected LN flare or for persistent or worsening disease despite appropriate treatment (1). Histologic classification remains critical because clinical findings alone cannot reliably distinguish proliferative from membranous LN.

When kidney biopsy cannot be safely performed because of a high bleeding risk including thrombocytopenia, markedly reduced kidney function, or antiphospholipid syndrome, treatment must be guided by the clinical presentation and available laboratory findings, with nephritic features suggesting proliferative LN and nephrotic-range proteinuria with hypoalbuminemia raising concern for membranous LN. However, these clinical patterns should not be considered substitutes for histologic classification. KDIGO and EULAR similarly emphasize biopsy-based diagnosis, classification, assessment of activity and chronicity, and treatment selection (2, 3).

The 2024 ACR guideline lowers the threshold for kidney biopsy to a UPCR >0.5 mg/mg compared with >1.0 mg/mg in the 2012 guideline (1, 14). This lower threshold is consistent with recommendations from both the current KDIGO and EULAR (2, 3). However, unlike KDIGO and EULAR, which also list an active urinary sediment or glomerular hematuria as an indication for biopsy, the 2024 ACR guideline does not include urinary sediment findings among its biopsy criteria. The 2012 ACR guideline had defined hematuria as ≥5 red blood cells per high-power field, and indicated biopsy when it accompanied proteinuria ≥0.5 g/24 h (14). In practice, some pediatric nephrologists consider kidney biopsy when UPCR is >0.2 mg/mg, particularly when accompanied by active urinary sediment, hematuria, hypertension, hypocomplementemia, rising anti-dsDNA, or declining kidney function (15).

In children, silent LN is well recognized, and clinically significant Class III, IV, or V disease may occur despite minimal or absent urinary abnormalities. Pediatric cohorts have reported substantial rates of biopsy-proven silent LN, including proliferative disease. In a single-center pediatric cohort of 69 children with LN, 22 had silent disease (16). A systematic review of 37 studies describing 639 patients with silent LN, spanning ages 2 to 70 years, found Class III, IV, or V lesions in approximately 30% (17). Some pediatric centers, particularly in Japan, have therefore adopted a more proactive baseline-biopsy approach in children with newly diagnosed SLE; in one such series of 45 children undergoing baseline biopsy, silent LN was identified in 25 (55.5%), of whom 6 had Class III disease (18). The potential advantage is earlier detection of treatable renal disease; however, this approach must be balanced against biopsy-related risks, and there is currently no prospective evidence that universal baseline biopsy improves long-term renal outcomes. Thus, whether a child with clinically silent disease should undergo baseline biopsy remains an area of uncertainty and should be individualized according to the overall clinical and serologic risk profile. In both pediatric series, low C3 and C4 levels were associated with proliferative lesions among children with silent disease, suggesting that hypocomplementemia may be a practical trigger for considering biopsy when urinary findings are minimal (16, 18).

### 3.3 Histological classification

The 2024 ACR guideline anchors histologic classification in the 2018 revision of the International Society of Nephrology/Renal Pathology Society (ISN/RPS) classification, which clarified lesion definitions and incorporated modified National Institutes of Health (NIH) activity and chronicity indices (19). The six-class classification system is the same in children and adults, as is the characteristic “full-house” immunofluorescence pattern, with deposition of IgG, IgA, IgM, C3, and C1q (Table 2) (8, 19). Proliferative LN (Class III/IV), alone or in combination with Class V, accounts for approximately 50%–70% of pediatric LN biopsies (7). The 2024 ACR guideline did not reach sufficient consensus to make treatment recommendations for Class II LN or thrombotic microangiopathy (TMA) in the setting of SLE. For lupus podocytopathy, evidence remains limited, and optimal treatment is not well established (1). Recent pediatric work suggests that the scoring of certain chronic lesions, in particular the proportion of fibrous crescents and interstitial fibrosis and tubular atrophy (IFTA), can be adjusted to better discriminate kidney outcomes in children (12).

**Table 2:** ISN/RPS 2018 classification of lupus nephritis (19).

LN Class	Name	Defining features
I	Minimal mesangial LN	Normal glomeruli on light microscopy; mesangial immune deposits on immunofluorescence
II	Mesangial proliferative LN	Mesangial hypercellularity with mesangial immune deposits
III	Focal LN	Focal glomerulonephritis involving <50% of glomeruli
IV	Diffuse LN	Diffuse glomerulonephritis involving ≥50% of glomeruli
V	Membranous LN	Subepithelial immune deposits
VI	Advanced sclerotic LN	≥90% globally sclerosed glomeruli without residual activity

The 2018 revision eliminated the class IV-S and IV-G subdivisions and replaced the active (A) and chronic (C) designations for classes III and IV with activity and chronicity indices scored across all classes.

## 4 Treatment of lupus nephritis

### 4.1 General management

Regarding lupus nephritis treatment, the 2024 ACR guideline emphasizes two overarching goals: early diagnosis and treatment, and prevention of chronic kidney damage. As Good Practice Statements, the 2024 ACR guideline recommends prompt kidney biopsy when lupus nephritis is suspected and initiation of glucocorticoid treatment while awaiting biopsy and histopathology results to avoid delays in treatment (1). To preserve long-term kidney function, treatment focuses on achieving and maintaining renal response, preventing disease flares, and minimizing cumulative treatment-related toxicity. Supportive management includes control of proteinuria and blood pressure, with attention to modifiable cardiovascular and metabolic risk factors such as excess weight (1).

In addition, the ACR outlines several key principles for lupus nephritis management. Medication doses should be adjusted according to kidney function throughout treatment, particularly in patients with reduced GFR. Hydroxychloroquine is strongly recommended for all patients with LN unless contraindicated, given its benefits in reducing disease activity and flares and improving long-term outcomes. RAAS inhibition is conditionally recommended for patients with any elevation in proteinuria, including proteinuria below 0.5 mg/mg, to reduce proteinuria and help preserve kidney function. Optimal care also requires close collaboration between Rheumatology and Nephrology, with shared decision-making among clinicians, patients, and families to individualize treatment according to disease severity, comorbidities, treatment goals, and patient preferences (1). Healthcare disparities may adversely affect outcomes in children with lupus nephritis. Equitable implementation of evidence-based treatment recommendations is essential to ensure timely diagnosis, appropriate treatment, and improved long-term outcomes across diverse pediatric populations (1).

### 4.2 What has changed between 2012 and 2024? From dual therapy to triple therapy for proliferative LN

The largest conceptual shift in the 2024 ACR guideline is the move from sequential induction and maintenance therapy to upfront triple therapy with continuous combination treatment for proliferative lupus nephritis (LN) (Table 3). The 2012 ACR guideline, developed for adults, used distinct induction and maintenance phases: pulse intravenous methylprednisolone followed by oral glucocorticoids at 0.5–1 mg/kg/day, combined with either mycophenolate mofetil (MMF) or cyclophosphamide (CYC) for induction, followed by MMF or azathioprine with lower-dose glucocorticoids for maintenance (14).

**Table 3:** Evolution of treatment for class III/IV LN: 2012 versus 2024 ACR (1, 14).

Feature	2012 ACR (adults)	2024 ACR (adults and children)
Overall strategy	Sequential dual therapy (induction then maintenance)	Upfront triple therapy; induction/maintenance distinction blurred (ongoing therapy)
Glucocorticoids	Pulse IV methylprednisolone, then oral 0.5–1 mg/kg/day	Pulse IV methylprednisolone, then oral ≤0.5 mg/kg/day (max 40 mg), tapered to ≤5 mg/day by 6 months
Immunosuppression	MMF or CYC (high- or low-dose) for induction; MMF or azathioprine for maintenance	MMF (preferred) or low-dose (Euro-Lupus) CYC, plus a third agent: belimumab or a CNI
Maintenance oral glucocorticoid	Approximately 10 mg/day	Continued taper to ≤5 mg/day
Response assessment	Approximately 6 months	Up to 12 months

In contrast, the 2024 ACR guideline conditionally recommends initiating triple therapy for most patients with Class III/IV LN, with or without Class V: pulse intravenous glucocorticoids followed by a lower-dose oral glucocorticoid taper [0.5 mg/kg/day (max 40 mg)], plus a mycophenolic acid analog (MPAA) or low-dose CYC, and a third agent, either belimumab or a calcineurin inhibitor (CNI). These therapies are intended as ongoing combination treatment rather than as separate induction and maintenance phases. The guideline conditionally recommends continuing immunosuppressive therapy for at least 3–5 years in patients who achieve a complete renal response (1).

The shift to triple therapy is based largely on adult trials. BLISS-LN showed that adding belimumab to standard therapy improved renal responses over two years, while AURORA-1 demonstrated improved one-year complete renal response with voclosporin added to MMF and low-dose steroids (20, 21). The Euro-Lupus Nephritis Trial established low-dose CYC as an effective, less toxic alternative to high-dose CYC and provided the basis for the dosing used in current guidelines (22). Importantly, none of these trials enrolled children.

Two features of the new approach are particularly relevant to pediatric practice. First, the emphasis on glucocorticoid minimization is especially important in growing children, who face greater long-term consequences from cumulative steroid exposure. Second, the 2024 guideline allows up to 12 months to assess renal response, recognizing that many patients do not achieve remission by six months (1). However, the optimal dosing and long-term safety of the third agents in children remain uncertain and are discussed below.

### 4.3 Treatment targets and response definitions

The 2024 ACR guideline sets the treatment goal as a complete renal response (CRR) or at least a partial renal response (PRR) within 6–12 months, and it defines these states precisely (1). A complete response requires a UPCR below 0.5 mg/mg with stable or improved kidney function (within about 20% of baseline); a partial response requires at least a 50% reduction in proteinuria with a UPCR below 3 mg/mg and stable function. Nonresponse is the failure to reach at least a PRR by 6–12 months, and refractory disease is persistently active LN without at least a PRR after two adequate, sequential 6-month courses. The guideline conditionally recommends early treatment escalation, particularly within 3 months, when kidney function is rapidly declining or proteinuria is increasing, and continue immunosuppression for at least 3–5 years after complete remission (1). In pediatric practice, some nephrologists aim for a lower UPCR target (<0.2 mg/mg) as the goal for complete remission, although this is more stringent than the ACR definition (9).

## 5 Treating class III/IV lupus nephritis (with or without class V)

For proliferative LN, whether new-onset or a flare, the 2024 ACR guideline **strongly recommends hydroxychloroquine** for patients with lupus nephritis unless contraindicated and **conditionally recommends RAAS inhibition** for elevated proteinuria. For active Class III/IV lupus nephritis, with or without Class V disease, the guideline **conditionally recommends triple therapy** consisting of glucocorticoids plus an immunosuppressive agent and either belimumab or a calcineurin inhibitor (1). Glucocorticoids are given as pulse intravenous methylprednisolone (250–1000 mg daily for 1–3 days) followed by oral glucocorticoids at no more than 0.5 mg/kg/day (maximum 40 mg), tapered to 5 mg/day or less by six months. The clinician then chooses between an MMF-based pathway (MMF plus belimumab, or MMF plus a CNI) and a CYC-based pathway (Euro-Lupus CYC plus belimumab, with MMF added after the CYC course is complete) (1). The guideline does not specify a particular CNI, given limited comparative data, although voclosporin is the agent with a dedicated phase 3 LN trial and an adult regulatory approval (21).

The most useful part of the guideline is its logic for choosing the third agent. Only part of that logic is a graded recommendation. For patients with moderate-to-severe extrarenal manifestations, the guideline conditionally recommends a triple regimen containing belimumab over one containing a CNI; conversely, for patients with proteinuria of 3 g/g or more, it conditionally recommends a regimen containing a CNI over one containing belimumab (1). The quantitative thresholds often quoted alongside these, namely an eGFR of approximately 45 mL/min/1.73 m^2^ and a blood pressure above 165/105 mmHg favoring belimumab, together with significant chronicity on biopsy, appear in the narrative accompanying that recommendation rather than among the graded recommendations themselves, and reflect the eligibility boundaries of the adult trials on which the guideline rests (1). Their limited applicability to children is discussed below. Low-dose CYC is reserved for specific situations: patient preference, intolerance or adherence concerns, or rapidly progressive glomerulonephritis (1).

This framework translates conceptually to children, but its quantitative thresholds remain largely adult-derived. The ACR criteria for selecting belimumab versus a calcineurin inhibitor (CNI) require a particular caution when applied to children. The suggested eGFR threshold of approximately 45 mL/min/1.73 m^2^ and blood pressure threshold of >165/105 mmHg are derived primarily from adult evidence and have not been validated as pediatric thresholds (1). In children, treatment selection should instead incorporate age- and sex-appropriate blood pressure percentiles, kidney function relative to age and baseline, degree of proteinuria, biopsy findings, extrarenal disease activity, and the potential renal and metabolic toxicity of CNIs. Thus, these ACR thresholds should be viewed as clinical guidance rather than fixed pediatric cutoffs, with treatment individualized according to disease severity and patient-specific factors. There is a need for pediatric-specific consensus recommendations to guide treatment selection and define appropriate thresholds for children with lupus nephritis. Belimumab is approved for children with SLE aged 5 years and older, and intravenous belimumab has been approved for pediatric LN since 2022. Direct pediatric safety, pharmacokinetic, and efficacy data are available, but LN-specific evidence remains extrapolated: the PLUTO trial excluded acute severe LN, and no randomized trial of belimumab in pediatric LN has been performed (3, 23). In contrast, experience with calcineurin inhibitors (CNIs), particularly in young children with LN, remains more limited (1, 3). Voclosporin is not approved for children with LN (8, 13).

Response is assessed at 6–12 months and drives the next step. A complete response is followed by the same triple therapy for 3–5 years, with MMF preferred over azathioprine for maintenance. A partial response, with improving proteinuria and stabilizing eGFR, also continues triple therapy with steroid tapering and individualized adjustment. Failure to reach at least a partial response prompts an adherence check first, then a switch to an alternative triple-therapy regimen that may include an anti-CD20 agent such as rituximab or obinutuzumab. Persistent failure after a second course triggers another adherence check, consideration of a repeat biopsy (to look for a second diagnosis or advanced chronicity), addition of anti-CD20 therapy, combination regimen (MMF, belimumab, and a CNI together), or referral to a clinical trial (1). The obinutuzumab signal is no longer hypothetical. The phase 2 NOBILITY trial showed benefit when obinutuzumab was added to MMF and steroids, and the phase 3 REGENCY trial then showed a superior renal response with obinutuzumab in active class III/IV LN (24, 25). The guideline anticipates that obinutuzumab will feature in future recommendations (1).

The pediatric evidence for the backbone agents is limited but meaningful. A randomized trial in adolescents found MMF to be at least as effective as intravenous CYC for induction, with a comparable safety profile, providing rare direct pediatric evidence supporting an MMF-based approach (26). In contrast, evidence for the newer third agents is largely extrapolated from adult trials.

## 6 Treating pure class V lupus nephritis

Pure membranous LN follows a parallel but distinct treatment pathway. The 2024 ACR guideline strongly recommends hydroxychloroquine and conditionally recommends RAAS inhibition for any elevation in proteinuria, then stratifies therapy by proteinuria level. For UPCR ≥1 mg/mg, the guideline conditionally recommends triple therapy with glucocorticoids, MMF, and CNI; for UPCR <1 mg/mg, it conditionally recommends glucocorticoids and/or a single immunosuppressive agent (MMF, azathioprine, or a CNI) (1). Treatment response is assessed similarly to proliferative LN, with immunosuppression continued for 3–5 years after complete renal response. For inadequate response, adherence should be assessed and an alternative regimen considered, with repeat biopsy and combination therapy reserved for refractory disease (1).

The pediatric concerns are similar to those in proliferative LN, with one important difference: pure Class V disease is less common and consequently even less studied in children**.** The emphasis on CNIs for heavier proteinuria is supported by their mechanism of action and adult trial data, but their long-term metabolic and nephrotoxic effects in children requiring prolonged therapy remain uncertain.

## 7 The sharpest translation gap: glucocorticoid dosing

Across the three contemporary guidelines, the direction of glucocorticoid therapy is consistent: start promptly with higher doses, but taper rapidly, as triple therapy makes lower cumulative steroid exposure possible without compromising renal outcomes (1–3). This principle is particularly important in pediatrics, where glucocorticoid toxicity extends beyond bone and metabolic effects to impaired linear growth and delayed puberty. However, the quantitative recommendations differ, and most are not specifically designed for children (Table 4). The 2024 ACR guideline conditionally recommends an initial oral glucocorticoid dose of 0.5 mg/kg/day (maximum 40 mg/day), tapering to a target of 5 mg/day by six months (1). KDIGO 2024 provides a severity-based approach, with initial doses of approximately 0.5–1.0 mg/kg/day (maximum 40–80 mg/day) followed by a structured taper (2). EULAR 2025 recommends an initial oral dose of 0.3–0.7 mg/kg/day in adults and, importantly, specifies a separate starting dose of 0.6–1.0 mg/kg/day for pediatric patients, noting that weight-based prednisone dosing remains the norm in pediatric practice (3). The pediatric range endorsed by EULAR is therefore approximately double the ACR ceiling of 0.5 mg/kg/day, and no pediatric trial adjudicates between them. Thus, clinicians treating children must reconcile these adult-derived recommendations with the child's weight, growth trajectory, disease severity, and susceptibility to glucocorticoid toxicity.

**Table 4:** Glucocorticoid dosing across guidelines and pediatric consensus (1–3, 27, 28).

Parameter	2024 ACR	2024 KDIGO	2025 EULAR	Pediatric consensus
IV methylprednisolone pulse	250–1000 mg/day for 1–3 days	250–500 mg/day for 1–3 days	500–2500 mg cumulative, given over 1–3 days, before oral glucocorticoid	CARRA: standardized oral, mixed, or IV-pulse options
Initial oral glucocorticoid (prednisone-equivalent)	≤0.5 mg/kg/day (max 40 mg)	Approximately 0.5–1.0 mg/kg/day by severity (reduced-dose scheme 0.5–0.6, max 40; moderate-dose 0.6–0.7, max 50; high-dose 0.8–1.0, max 80)	0.3–0.7 mg/kg/day (approximately 0.6–1.0 mg/kg/day in children)	No pediatric-specific dose in ACR; Chalhoub SSR individualizes dose to proteinuria, eGFR, disease activity, and time since biopsy
Taper target	≤5 mg/day by 6 months	To ≤5 mg/day (examples reach 5 mg/day at about weeks 11/17/21 for low/moderate/high starting doses); lowest possible during maintenance	≤5 mg/day by 4–6 months	Target the lowest effective dose to limit growth delay and hypertension

Genuinely pediatric evidence on glucocorticoid dosing exists outside the adult-derived guidelines. The Standardized Steroid Dosing Regimen (SSR) developed by Chalhoub and colleagues was based on pediatric patient profiles and physician consensus, with dosing tailored to proteinuria, eGFR, disease activity, and time since biopsy (27). The Childhood Arthritis and Rheumatology Research Alliance (CARRA) consensus treatment plans describe similar standardized induction glucocorticoid regimens for newly diagnosed proliferative LN in juvenile SLE, including predominantly oral, mixed, and predominantly intravenous approaches (28). These resources directly address pediatric steroid dosing but are separate from the 2024 ACR guideline. In practice, combining the ACR framework with pediatric-specific tools such as the SSR and CARRA regimens may provide a more appropriate approach to glucocorticoid dosing in children.

## 8 Monitoring disease activity

Monitoring is one area where translation from adult to pediatric practice is relatively straightforward. The 2024 ACR guideline strongly recommends quantifying proteinuria at least every three months in patients who have not achieved complete renal response and every 3–6 months once sustained clinical renal remission is established. Serum complement levels and anti–double-stranded DNA antibodies should be assessed at each clinic visit, but not more frequently than monthly (1). In pediatric practice, blood pressure, BMI, height, and medication adherence are routinely assessed at every visit and are particularly important for capturing treatment- and disease-related complications in growing children. Thus, monitoring frequency is an area in which adult and pediatric practice largely align.

## 9 Pediatric-specific considerations the guideline addresses only partially

To its credit, the 2024 ACR guideline incorporates several pediatric considerations, all issued as ungraded Good Practice Statements: pediatric-appropriate glucocorticoid regimens to limit cumulative exposure, monitoring for delayed pubertal onset and decreased growth velocity with referral to pediatric endocrinology if indicated, fertility preservation for patients receiving intravenous CYC, and a structured, intentional transition to adult care (1). These are important recommendations, but two areas require greater attention. Fertility preservation is a particularly important and modifiable concern with CYC exposure. The Euro-Lupus low-dose CYC regimen reduces cumulative exposure and may therefore help mitigate gonadal toxicity, a consideration that should be explicitly incorporated into pediatric treatment decisions (22). Transition to adult care is similarly more than a single transfer event. Adolescents and young adults with cSLE and LN are at risk for disease flares and medication nonadherence during transition, and structured, individualized transition planning may improve long-term outcomes (7, 29). Thus, simply recommending a “transition plan” understates the complexity of this process; pediatric literature increasingly recognizes transition as a distinct clinical competency requiring longitudinal preparation and support (29).

## 10 Where does the 2024 ACR guideline run out of pediatric evidence?

The guideline is candid about its limitations. First, long-term safety data for biologic therapies in children remain limited. Belimumab and anti-CD20 therapies are increasingly incorporated into the treatment algorithm, but pediatric data over the decades-long time horizon relevant to children are lacking, leaving many recommendations dependent on adult evidence (1, 8). Second, pediatric glucocorticoid dosing remains insufficiently defined and often relies on adult-derived recommendations (1). Third, and underlying both limitations, is the lack of pediatric randomized controlled trials. Children have historically been excluded from pivotal LN trials, making extrapolation from adult data unavoidable (8).

The SHARE recommendations were developed in part to address this gap from a pediatric-first perspective. Compared with adult-derived guidelines, they place greater emphasis on growth, development, treatment toxicity, and family-centered care (4, 5).

In short, the 2024 ACR guideline represents important progress by explicitly incorporating children, but much of its evidence base remains adult derived. Pediatric-specific details therefore still need to be supplied by resources such as the SSR, CARRA treatment plans, SHARE recommendations, and clinical judgment.

## 11 Discussion

Reading the 2024 ACR guideline through a pediatric lens reveals a consistent pattern: the conceptual framework translates well, but the operational details do not always. Early biopsy, the ISN/RPS classification system, multi-target immunosuppression, response-driven escalation, and prolonged maintenance are fundamental principles that apply across age groups. In contrast, the numerical parameters (prednisone dosing, eGFR and blood-pressure thresholds for selecting belimumab versus a CNI, and proteinuria thresholds for defining response) are largely derived from adult data and may not fully account for pediatric physiology, pharmacokinetics, growth, and the much longer treatment horizon.

Two practical conclusions follow. First, the 2024 ACR guideline is best used as a framework rather than a formulary in pediatric practice. Its therapeutic principles and response-based strategy can be adopted, while drug dosing and other quantitative thresholds should be reconciled with pediatric-specific resources, including the SSR and CARRA treatment plans, and individualized to the child (27, 28). Second, where evidence is extrapolated, clinicians should prioritize close surveillance and minimizing cumulative treatment toxicity, particularly glucocorticoid exposure. The modern shift toward lower steroid exposure across ACR, KDIGO, and EULAR further supports this approach (1–3).

The role and timing of therapeutic plasma exchange (TPE) in children with severe SLE/LN warrant specific consideration because of the potentially aggressive disease course and limited pediatric evidence. TPE is not part of routine therapy for lupus nephritis and is generally reserved for specific complications such as thrombotic thrombocytopenic purpura (TTP) and catastrophic antiphospholipid syndrome (CAPS) (1, 3). The 2024 ACR guideline does not provide specific recommendations for SLE-associated thrombotic microangiopathy (TMA) or antiphospholipid nephropathy. Pediatric evidence is limited to small case series describing the use of TPE in severe or refractory SLE, including cases complicated by TTP, diffuse alveolar hemorrhage, or severe crescentic glomerulonephritis (30). Where a pediatric-specific position has been taken, it is more conservative rather than more aggressive: KDIGO advises that adults at intermediate-to-high risk of TTP should begin plasma exchange while awaiting confirmatory testing, whereas in children, because TTP is less common and plasma exchange has been associated with considerable morbidity, it is acceptable to defer the procedure for 24 to 48 h until the ADAMTS13 result is available (2). Early multidisciplinary consideration of TPE remains appropriate in selected life-threatening presentations, such as diffuse alveolar hemorrhage or rapidly progressive crescentic disease, but there is currently no evidence to support adopting a lower threshold or earlier timing in children than in adults. Decisions should therefore be individualized according to the clinical presentation and underlying complication.

This review has several limitations. It is a narrative synthesis centered on the 2024 ACR guideline and a pediatric nephrology perspective rather than a systematic review, and it does not formally grade the evidence supporting each recommendation. Much of the pediatric epidemiologic literature derives from cohort studies and expert reviews with heterogeneous populations and definitions; therefore, reported ranges for LN frequency, flares, remission, and CKD should be interpreted as representative rather than pooled estimates (7, 9–11). The therapeutic landscape is also evolving rapidly. Obinutuzumab now has phase 3 adult data and is already included in the 2025 EULAR recommendations, although it is not yet incorporated into the ACR guideline; emerging therapies such as anifrolumab and cellular therapies will raise similar questions about translating adult evidence to children (3, 25). Ultimately, because children remain underrepresented in pivotal LN trials, many pediatric recommendations remain necessarily inferential. Closing this evidence gap should be a priority for the next generation of lupus nephritis trials and guidelines.

## 12 Future directions

Several developments could help narrow the pediatric evidence gap. The anti-CD20 antibody obinutuzumab, supported by the NOBILITY and REGENCY trials in adults with Class III/IV LN, has already been incorporated into the 2025 EULAR recommendations as a first-line combination partner for mycophenolate (grade 1b/A) and is to feature in future ACR guidance, making timely pediatric studies particularly important (1, 3, 24, 25). Other emerging therapies, including type I interferon blockade and cellular therapies, will raise similar questions about translating adult evidence to children. On the diagnostic side, noninvasive biomarkers of renal activity and damage, such as urinary CD163, may eventually reduce the need for repeat biopsy and are being studied in pediatric populations (7, 12). Most importantly, children should be included in LN trials from the outset, or pediatric bridging studies should be conducted alongside adult drug development and approvals. This would allow future recommendations to be based on pediatric evidence rather than extrapolation (8). Harmonizing ACR, KDIGO, and EULAR treatment frameworks with pediatric resources such as the SSR and CARRA regimens could also provide clinicians with a more coherent, pediatric-specific treatment approach.

## 13 Conclusion

The conceptual architecture of modern lupus nephritis care, including early biopsy, triple therapy, steroid minimization, response-driven escalation, and prolonged maintenance, translates well from adults to children. The quantitative details do not. Glucocorticoid dosing, the long-term safety of biologics, and the developmental consequences related to fertility, growth, and transition remain distinctly pediatric questions that adult trials cannot adequately answer. These are precisely the areas where the 2024 ACR guideline relies on adult evidence or provides limited guidance. Until children are studied directly, clinicians should use the guideline for its principles, supplement it with available pediatric evidence, and view extrapolated treatment parameters as informed estimates rather than established pediatric standards.
